# Stable isotope‐derived measurements of protein synthesis detect amino acid‐induced anabolism in skeletal muscle microtissues not captured by puromycin labelling

**DOI:** 10.1113/EP094100

**Published:** 2026-10-11

**Authors:** Yekaterina Tiper, Cassidy T. Tinline‐Goodfellow, Penney M. Gilbert, Daniel R. Moore

**Affiliations:** ^1^ Institute of Biomedical Engineering University of Toronto Toronto Ontario Canada; ^2^ Donnelly Centre University of Toronto Toronto Ontario Canada; ^3^ Faculty of Kinesiology and Physical Education University of Toronto Toronto Ontario Canada; ^4^ Department of Cell and Systems Biology University of Toronto Toronto Ontario Canada

**Keywords:** anabolic stimuli, engineered skeletal muscle, protein synthesis, puromycin labelling, stable isotope tracers

## Abstract

Skeletal muscle microtissues are valuable in vitro models for studying the stimuli regulating muscle protein synthesis (MPS), the key determinant of changes in muscle mass. Differentiated between opposing posts, microtissues contain aligned, contractile myotubes, providing a controlled system for investigating the responses of skeletal muscle to nutrient and contractile stimulation. However, microtissue MPS responses to these stimuli remain under‐characterized. Stable isotope‐labelled amino acid tracers deliver sarcoplasmic and myofibrillar fractional synthetic rates (FSR) for MPS in human studies but have not been implemented in engineered skeletal muscle. We close these gaps by characterizing amino acid (AA), ketone and contraction‐induced MPS in 3D microtissues and 2D myotubes (without contraction) derived from the same primary myoblast line, using stable isotope tracers and puromycin incorporation. Following 4 h of AA withdrawal microtubes and myotubes underwent 2 h of acute AA and/or ketone treatment (±contraction in microtissues). In microtissues, sarcoplasmic FSR increased significantly during amino acid re‐addition (*P *< 0.0001), whereas myofibrillar FSR displayed a main effect of condition (*P = *0.0018), with an overall lesser magnitude of change compared to the sarcoplasmic fraction. However, both fractions were unresponsive to ketone stimulation, and the selected contraction regime (when combined with ketones and/or AA; all *P* ≥ 0.985). 2D myotubes showed significant increases in sarcoplasmic and myofibrillar FSR in response to amino acid treatment (both *P* ≤ 0.0003). Notably, microtissues demonstrated a more stable myofibrillar protein fraction, with a sarcoplasmic‐to‐myofibrillar FSR ratio of ∼2:1 which closely resembled that of native human muscle. The puromycin‐based approach only detected subtle differences in 2D myotubes (main effect *P = *0.031) with AA provision being greater than ketones (*P = *0.0196) with no other *post hoc* differences (all *P* ≥ 0.221). Puromycin failed to detect MPS responses to any stimulus in microtissues (all *P* ≥ 0.834), highlighting the greater sensitivity of tracer‐based measurements, particularly where longer time scales are needed to capture an effect. These findings support the use of engineered muscle and isotope‐derived measurements of MPS in future studies of stimuli regulating skeletal muscle mass.

## Abstract (highlights)

- **What is the central question of this study?**
  Stable isotope tracers are emerging as a powerful approach to measure fraction‐specific protein synthesis: what is their efficacy relative to conventional puromycin labelling and can they be applied to engineered skeletal muscle?
- **What is the main finding and its importance?**
  By implementing stable isotope tracers in engineered muscle, the ability to capture anabolic responses that are undetected by puromycin‐based methods was showcased. The myofibrillar protein fraction of microtissues is more stable than the sarcoplasmic fraction, a property of native muscle, absent in 2D myotubes. These findings demonstrate the physiological relevance of engineered muscle and support the adoption of isotope‐derived measurements in future studies.

## 1 INTRODUCTION

Skeletal muscle plays a critical role in overall health, facilitating movement, whole‐body protein metabolism (McKendry et al., 2021) and glucose homeostasis (Merz & Thurmond, 2020). Maintaining muscle mass throughout life is essential, as age‐related muscle loss, or sarcopenia, can lead to increased frailty and a higher risk of falls and fractures (Yeung et al., 2019). Muscle protein synthesis (MPS) is the primary determinant of net muscle balance, more so than muscle protein breakdown (MPB). The most potent anabolic stimuli of MPS are amino acid ingestion and muscle contraction (i.e. resistance exercise) (Burd et al., 2009). In recent years, ketone bodies, particularly β‐hydroxybutyrate and acetoacetate, have also gained increasing interest for their role in enhancing MPS (Hannaian et al., 2024). However, human clinical trials can be costly, invasive and logistically challenging, which has led to the exploration of cell‐based models to estimate the in vivo effects of nutrients and/or contraction.

Studies using two‐dimensional (2D) myotube cultures in vitro have advanced our understanding of the molecular mechanisms that regulate skeletal muscle mass, particularly the balance between MPS and MPB (Gran & Cameron‐Smith, 2011; Huang et al., 2007; Rommel et al., 2001). These models eliminate many of the confounding factors present in vivo, such as fluctuating hormone levels and immune system responses, while offering greater experimental control over variables like dosage, nutrient availability and treatment duration. Additionally, they enable higher‐throughput experimentation due to their simpler handling and scalability compared to in vivo studies. However, 2D myotube cultures are limited in their ability to model native muscle structure and function, as they lack alignment and tend to detach from the substrate upon contraction. To overcome these limitations, three‐dimensional (3D) models of skeletal muscle have emerged (Hofemeier et al., 2021; Madden et al., 2015; Vandenburgh et al., 2008). These models involve the differentiation of myoblasts in an extracellular matrix under uniaxial tension between two fixed points. Unlike 2D systems, 3D skeletal muscle models reproduce key aspects of native muscle architecture, support prolonged culture durations, and facilitate studies of contractile stimuli, while retaining the advantages of in vitro experimentation (Khodabukus, 2021). To fully harness the potential of 3D models in investigating skeletal muscle mass regulation, reliable methods for measuring MPS in these models are required.

Puromycin is a widely used tool to measure MPS rates in vitro. This aminonucleoside antibiotic, produced by the bacterium *Streptomyces alboniger*, acts as a structural analogue of aminoacyl tRNAs. Puromycin is incorporated into nascent peptide chains but prematurely terminates their elongation due to its non‐hydrolysable amide bond (Aviner, 2020). When used in minimal amounts over 5–30 min pulses, puromycin incorporation into newly synthesized proteins reflects the rate of mRNA translation, which can be semi‐quantitatively assessed via immunoblotting of cell lysates (Schmidt et al., 2009). This technique, known as surface sensing of translation (SUnSET), is widely used in skeletal muscle research to measure protein synthesis both in vitro and in animal models (Goodman et al., 2011; Goodman, Kotecki et al., 2012; Goodman, Pierre et al., 2012).

Due to potential toxicity of puromycin, it is unsuitable for human use in research. In vivo human studies of MPS commonly rely on stable isotope‐labelled amino acid tracers (e.g., l‐[ring‐^2^H_5_]phenylalanine, l‐[1‐^13^C]leucine, etc.) administered orally or intravenously and quantitatively analysed via gas or liquid chromatography–mass spectrometry (Wilkinson et al., 2017). Stable isotope tracers, being chemically and functionally identical to their naturally occurring counterparts, allow amino acid kinetics to be traced over extended periods through the behaviour of the labelled compounds. In the tracer incorporation model, the rate of tracer incorporation into newly synthesized peptides is quantified over time, yielding fractional synthetic rates (FSR) as a measure of MPS (Kim et al., 2016). Studies investigating fraction‐specific MPS have demonstrated that the contractile myofibrillar pool (MyoFSR) is sensitive to both resistance exercise and amino acid ingestion, while the sarcoplasmic pool (SarcFSR) primarily responds to amino acid ingestion (Moore et al., 2009). Whether similar results may be observed in vitro is currently unknown. For example, stable isotope‐labelled amino acid tracers have been applied to 2D myotube systems (Atherton et al., 2009; Crossland et al., 2020), but contraction in these models is typically obtained through passive cell stretching or multi‐axial electrical pulse stimulation (Constantinou & Bastounis, 2023; Tarum et al., 2017). Consequently, fraction‐specific MPS has not been investigated in 3D systems over extended periods under conditions of nutrient availability and contractile stimulation. We previously developed MyoTACTIC, a 96‐well culture platform for the fabrication of human skeletal muscle microtissues (hMMTs) (Afshar et al., 2020; Lad et al., 2021). hMMTs are formed between a pair of opposing microposts in each well, with micropost deflection proportional to the force generated during contraction. In this study, we implement tracer to measure fraction‐specific MPS in hMMTs in response to amino acid, ketone and contractile stimulation. We compare stimulation‐induced MPS, as measured by both tracers and puromycin incorporation, in hMMTs to that of 2D myotubes derived from the same primary myoblast line. Our findings highlight the enhanced physiological relevance of engineered skeletal muscle compared to 2D myotubes and demonstrate the ability of tracer to capture anabolic responses that are undetected by puromycin‐based methods.

## 2 METHODS

### 2.1 Human primary myoblast culture and 2D differentiation

A human primary myoblast line was purchased from Cook MyoSite Inc. (SK‐1111‐P01358‐19F; Pittsburgh, PA, USA). The cell line was derived using a proprietary method from a sample of vastus lateralis muscle tissue of a 19‐year‐old Caucasian female with a body mass index of 24 and no known medical conditions. Myoblasts were passaged as previously described (Gulati et al., 2024; Tiper et al., 2025). Briefly, they were plated on collagen‐coated tissue culture dishes (Appendix Table A1) in growth medium (Table A1), which was replaced every other day. Myoblasts were passaged with trypsin once they reached ∼70% confluency and replated at a lower density. At passage 8, they were used for 2D myotubes and 3D tissue experiments. The population was ∼95% CD56^+^ at this passage, as determined by fluorescence‐activated cell sorting, indicating a high degree of myogenic purity.

For 2D differentiation, myoblasts were plated at a seeding density of ∼35,000 cells/cm^2^ on collagen‐coated six‐well plates (Table A1). The cells were cultured in growth medium without added basic fibroblast growth factor for 2 days before transitioning to 2D differentiation medium (Table A1). Half the medium was refreshed every other day. Anabolic responses of cultures were assessed on day 5 of differentiation.

### 2.2 MyoTACTIC culture platform fabrication and hMMT seeding

The polydimethylsiloxane (PDMS; Sylgard™ 184 silicone elastomer kit, Dow Corning; Midland, MI, USA) MyoTACTIC 96‐well culture platform was fabricated as previously described (Afshar et al., 2020; Lad et al., 2021). After fabrication, the plate was cut into portions containing 4 ± 2 wells. The PDMS portions were sonicated in isopropanol for 20 min, rinsed with dH_2_O, dried in a curing oven at 60–65°C, and then autoclaved in an instrument sterilization bag. Prior to use, a non‐adhesive surface was created by coating the portions with a 5% Pluronic® F‐127 (cat. no. P2443, Sigma‐Aldrich; St Louis, MO, USA) solution in dH_2_O overnight at 4°C. The solution was aspirated prior to hMMT seeding.

hMMTs were seeded in the MyoTACTIC platform as previously described (Afshar et al., 2020; Lad et al., 2021; Tiper et al., 2026). Primary myoblasts were resuspended in a hydrogel mixture (Table A1) at a concentration of 11.7 × 10^6^ cells/mL, with thrombin (cat. no. T6884, Sigma‐Aldrich; St Louis, MO, USA) added at 0.37 units/mg of fibrinogen (cat. no. F8630, Sigma‐Aldrich; St Louis, MO, USA) to induce clotting. The resulting hMMTs contained 175,000 cells/tissues. They were cultured in after‐seeding growth medium (Table A1) for 2 days before transitioning to 3D differentiation medium (Table A1). Half of the medium was refreshed every other day. Anabolic responses of hMMTs were assessed on day 14 of differentiation.

### 2.3 Anabolic and electrical stimulation conditions

For all conditions, 2D myotubes and 3D hMMTs were first subjected to a 4‐h anabolic knockdown in amino acid‐, horse serum‐ and insulin‐free medium. Following knockdown, the cultures were incubated for 2 h during the refeeding period in medium supplemented with either amino acids or ketones (4 mM β‐hydroxybutyrate [cat. no. 166898, Sigma‐Aldrich; St Louis, MO, USA] and 1.4 mM lithium acetoacetate [cat. no. A8509, Sigma‐Aldrich; St Louis, MO, USA]) (Vandoorne et al., 2017), or both amino acids and ketones (Table A1). A starvation condition was included as control, where myotubes and hMMTs underwent knockdown without nutrient stimulation. For 3D hMMTs, the effect of contraction on protein synthesis prior to nutrient stimulation was also measured. This involved conditions where hMMTs underwent an electrical stimulation protocol prior to incubation with amino acids and/or ketones. Electrical field stimulation (EFS) was applied to hMMTs between two needle electrodes placed on either side of the microtissue, as previously described (Afshar et al., 2020; Lad et al., 2021; Tiper et al., 2025). Twitch contractions were elicited using EFS at 1 Hz, 5 V and 60 ms, while tetanic contractions were elicited with EFS at 7 Hz, 5 V and 10 ms. The electrical stimulation regimen consisted of 20 twitch contractions followed by five tetanus contractions.

### 2.4 Protein synthesis measurements

During the 2‐h refeeding period or the final 2 h of starvation, 250 µM l‐[ring‐^2^H_5_]phenylalanine (cat. no. DLM‐1258‐5, Cambridge Isotopes; Tewksbury, MA, USA) was added to the medium of each hMMT to assess protein synthesis through tracer incorporation. Additionally, during the last 30 min of feeding or starvation, 1 µM of puromycin (cat. no. PUR333, BioShop; Burlington, ON, Canada) was included in the medium of each hMMT to measure protein synthesis via incorporation of puromycin into nascent peptides (Goodman et al., 2011). Immediately before cell lysis, an aliquot of medium was collected to measure precursor enrichment for tracer analysis, as described below. 2D myotubes and 3D hMMTs were collected after two washes in ice‐cold phosphate‐buffered saline. 2D myotubes were lysed and scraped in an ∼60 µL/well of Tris–TritonX buffer (20 mM Tris pH 7.2, 0.5% Triton X‐100), with three wells pooled per condition. 3D hMMTs were collected into pre‐chilled microtubes on ice with tweezers and then lysed in the Tris–TritonX buffer with mechanical homogenization using a tight‐fitting Teflon pestle.

Protein fractions of the homogenate were separated by centrifugation at 1500 *g* for 10 min at 4°C. The supernatant, containing soluble sarcoplasmic proteins, was transferred to a new microtube. The pellet, containing insoluble myofibrillar proteins, was rinsed by adding 500 µL of ice‐cold ddH_2_O, centrifuging at 1500 *g* for 10 min at 4°C , and discarding the resulting supernatant (Roberts et al., 2020). Myofibrillar proteins were then solubilized with the addition of 200 µL of 0.3 M NaOH, and heated at 50°C for 30 min, with vortexing every 10 min. Subsequently, 500 µL of 1 M perchloric acid was added to both the myofibrillar and sarcoplasmic protein tubes to precipitate proteins. The samples were centrifuged at 1600 *g* for 20 min at 4°C, and the supernatant was discarded. The resulting protein pellets were gently rinsed with ice‐cold 70% ethanol, centrifuging at 1600 *g* for 20 min at 4°C, and discarding the ethanol supernatant. Proteins were then hydrolysed into their constituent amino acids by incubation for ∼36 h at 110°C in 750 µL of 0.1 M HCl and 750 µL of Dowex resin (cat. no. Dowex50W‐X8–200, Supelco; Bellefonte, PA, USA) in 1 M HCl.

Amino acids were isolated over cation exchange columns constructed from glass wool in 5 mL syringes. The amino acids were eluted with 4 mL of 2 M NH_4_OH, dried down under a steady stream of nitrogen at 80°C, and resuspended in 200 µL of 0.1% formic acid. Liquid chromatography–tandem mass spectrometry (LC‐MS/MS) was then performed to analyse samples, monitoring mass‐to‐charge ratios (*m*/*z*) of 171.1/125 for l‐[ring‐^2^H_5_]phenylalanine, and 166.1/131 for natural phenylalanine, following established methods for human skeletal muscle (Hannaian et al., 2020) by the Analytical Facility for Bioactive Molecules at SickKids Research Centre (Toronto, ON, Canada).

For medium (i.e. precursor) enrichment analysis by GC‐MS, 50 µL of cell culture medium was mixed with 400 µL of acetonitrile and heptafluorobutyric anhydride‐derivatized identical to human plasma, as we have previously described (Hannaian et al., 2020).

Fractional synthetic rates (FSR) for both myofibrillar (MyoFSR) and sarcoplasmic (SarcFSR) fractions were calculated as previously described (Aguilera et al., 2025; Atherton et al., 2009) using the equation:

$$\mathrm{FSR}=\left[\left(\frac{E_{\mathrm{incorp}}}{E_{\mathrm{medium}}}\right)\times t^{-1}\right]\times 100$$

where *E*_incorp_ is the enrichment of protein‐bound l‐[ring‐^2^H_5_]phenylalanine, *E*_medium_ is the enrichment of the medium, and *t* is time (2 h in all experiments). FSR is expressed in percent per hour.

### 2.5 Immunoblotting

Immunoblotting for puroymcin was performed as we have previously described using a primary antibody directed towards puromycin (cat. no. MABE343, RRID: AB_2566826, Sigma‐Aldrich; St Louis, MO, USA) (Aguilera et al., 2025).

### 2.6 Statistical analysis

In 3D and 2D experiments, tracer and puromycin incorporation were analysed by pooling five to seven hMMTs or three wells from a six‐well plate, respectively, for each experimental condition. All experiments were performed on three separate occasions, constituting three biological replicates. Values are reported as means ± standard deviation (SD), and error bars in bar graphs represent SD. Statistical significance was determined using Welch's t‐test for comparison between two groups, and a one‐way ANOVA followed by Tukey's *post hoc* test for multiple group comparisons. Statistical significance was set to *P* ≤ 0.05 for all tests. Details of pooled tissue counts, means ± SD, statistical tests and *P*‐values for measurements of protein synthesis are provided in Appendix Table A2 (2D studies) and Appendix Table A3 (3D studies). Simple linear regression was performed to examine the relationships between variables. The *x*‐ and *y*‐axis metrics, *F*‐Statistic, *P*‐value, slope and coefficient of determination for each regression are provided in Appendix Table A4. All graphs were generated and statistical analyses were performed using GraphPad Prism 10.0 (RRID:SCR_002789; GraphPad Software, Boston, MA, USA).

## 3 RESULTS

### 3.1 Tracer provides greater sensitivity for measuring MPS than puromycin

To compare measurements of MPS derived from stable isotope tracers and puromycin, we assessed l‐[ring‐^2^H_5_]phenylalanine and puromycin incorporation during acute stimulation of 2D myotubes with amino acids, ketones (β‐hydroxybutyrate and lithium acetoacetate) or both, following anabolic knockdown (*n* = 3 wells per condition across *N* = 3 independent experiments; Figure 1a; Table A2). MPS in starved myotubes during knockdown served as the baseline. We found sarcoplasmic FSR increased significantly with amino acid treatment whereas ketone stimulation had no effect (*P = *0.0003 and *P *> 0.999, respectively; Figure 1b). Combined amino acid and ketone treatment significantly increased tracer‐derived sarcoplasmic FSR relative to starved myotubes but was not significantly different from amino acids alone (*P *< 0.0001 and *P = *0.255, respectively). A similar trend was observed for the myofibrillar FSR, which significantly increased with amino acid stimulation but remined unaffected by ketones (*P = *0.0002 and *P = *0.981, respectively; Figure 1c). Combined amino acid and ketone treatment significantly increased myofibrillar FSR relative to starved myotubes but again did not differ significantly from amino acids alone (*P *< 0.0001 and *P = *0.625, respectively). Linear regression analysis revealed a significant and strong positive relationship between myofibrillar and sarcoplasmic FSRs, with higher synthesis rates observed in the sarcoplasmic fraction across stimulation conditions (*R* ^2^ = 0.992, *m* = 0.707, *P *< 0.0001; Figure 1d). Puromycin incorporation demonstrated a main effect of condition (*P = *0.031), but *post hoc* pairwise comparisons revealed that compared to STV, incorporation was not significantly increased with amino acid, ketone or a combined nutrient stimulation (all comparisons *P* ≥ 0.220; Figure 1e,f). There was a significant difference between AA and Ketone conditions for puromycin incorporation (*P = *0.0196). This is consistent with a linear regression showing moderate non‐significant correlation between puromycin incorporation and tracer‐derived sarcoplasmic FSR (*R* ^2^ = 0.143, *m* = 0.631, *P = *0.225; Figure 1g). These results demonstrate the greater sensitivity conferred by a tracer‐based approach for quantifying MPS at longer time scales, as nutrient‐stimulated increases in protein synthesis were detectable using l‐[ring‐^2^H_5_]phenylalanine, which puromycin failed to capture.

**FIGURE 1:**
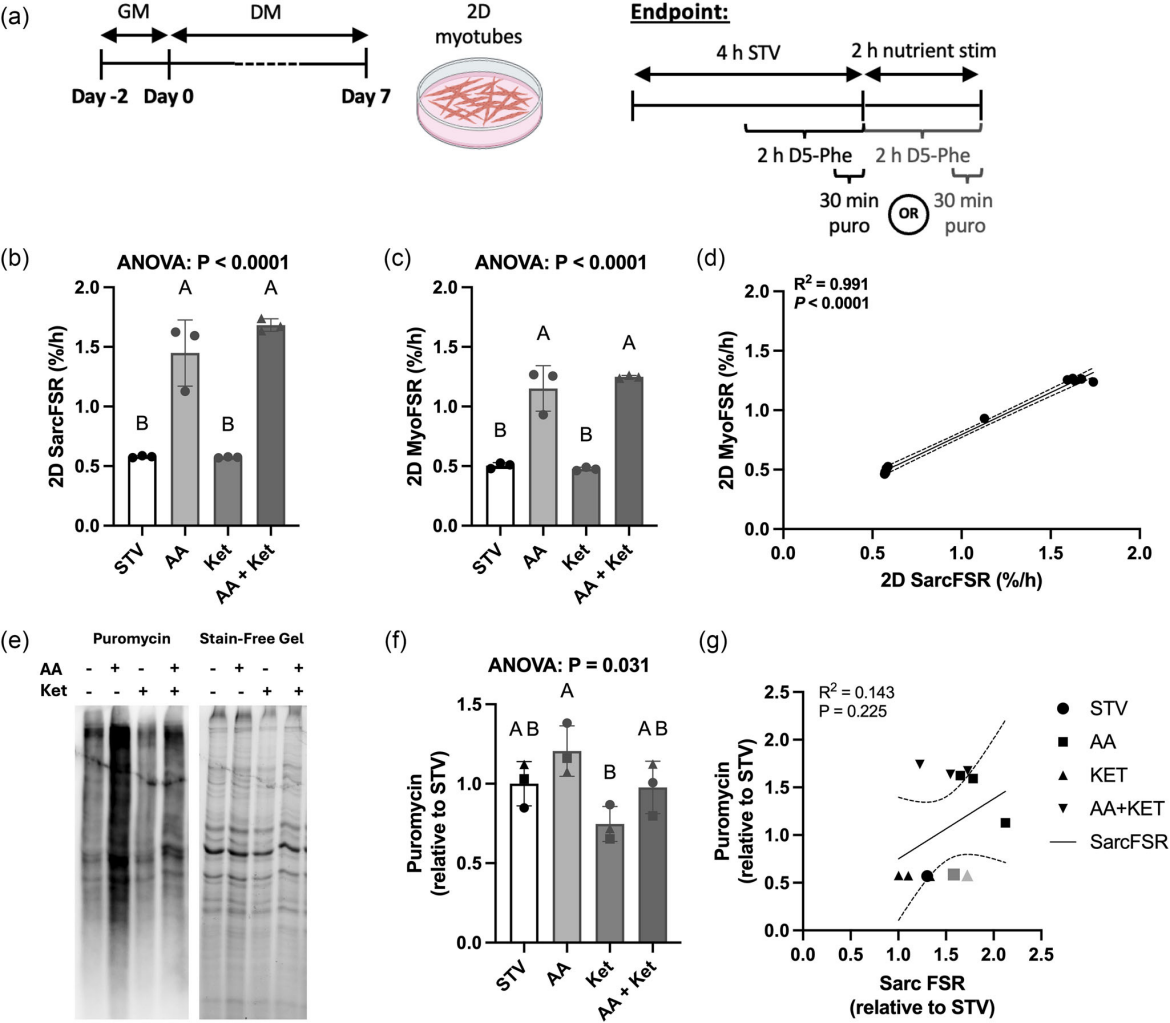
Stable isotope‐ versus puromycin‐derived measurements of nutrient stimulated muscle protein synthesis (MPS) in 2D myotubes. (a) Schematic overview of the timeline for 2D myotube culture and endpoint MPS measurements. Cells were cultured in growth medium (GM) for 2 days, followed by differentiation medium (DM) for 5 days. MPS was measured via incorporation of l‐[ring‐^2^H_5_]phenylalanine (D5‐Phe) and puromycin during anabolic knockdown (starved; STV), or stimulation with amino acids (AA), ketones (Ket), or both (AA + Ket) following knockdown. (b, c) Sarcoplasmic (Sarc; b) and myofibrillar (Myo; c) fractional synthetic rates (FSR) measured via D5‐Phe incorporation into sarcoplasmic and myofibrillar proteins, respectively. (d) Linear regression modelling the relationship between sarcoplasmic and myofibrillar FSR. (e) Representative image of western blot analysis for puromycin alongside the corresponding stain‐free total protein image. (f) Quantification of puromycin‐labelled peptides normalized to STV. (g) Linear regression modelling the relationship between sarcoplasmic FSR and puromycin incorporation. *n* = 3 wells pooled per condition across *N* = 3 independent experiments. Graphs display means ± SD. Circular, triangular, and square dots represent values from the first, second and third biological replicate, respectively. Statistical significance was determined using one‐way ANOVA followed by Tukey's *post hoc* test; different letters denote statistically different conditions (*P < *0.05).

### 3.2 Ketones and contraction do not enhance amino acid stimulated protein synthesis in hMMTs

To further compare the sensitivity of tracer‐ and puromycin‐based approaches for quantifying MPS and to investigate the combined effect of contractile and nutrient stimulation on MPS, we repeated the experiment in hMMTs with the inclusion of a contraction regimen (Figure 2aTable A3). l‐[ring‐^2^H_5_]Phenylalanine and puromycin incorporation were assessed during acute stimulation of hMMTs with amino acids, ketones or both, following anabolic knockdown with or without an accompanying electrical contraction protocol prior to treatment. Consistent with the results in 2D myotubes, we found tracer‐derived sarcoplasmic FSR increased significantly with amino acid treatment, whereas ketone stimulation had no effect (*P *< 0.0001 and *P *> 0.999, respectively; one‐way ANOVA; Figure 2b). Combined amino acid and ketone treatment significantly increased sarcoplasmic FSR relative to starved myotubes but was not significantly different from amino acids alone (*P *< 0.0001 and *P *> 0.999, respectively). Additionally, contractile stimulation alongside amino acid treatment significantly increased sarcoplasmic FSR relative to starved myotubes but did not enhance sarcoplasmic FSR beyond the level achieved with only amino acids (*P *< 0.0001 and *P *> 0.999, respectively; one‐way ANOVA). Increases in myofibrillar FSR (main effect *P = *0.0018) in response to amino acid stimulation, either alone (*P = *0.065) or in combination with ketones (*P = *0.052), contraction (*P = *0.080) or both (*P = *0.114), were not significant (Figure 2c). Despite the myofibrillar fraction being less responsive to anabolic stimuli, linear regression analysis revealed a strong, significant positive relationship between myofibrillar and sarcoplasmic FSRs (*R* ^2^ = 0.854, *m* = 0.326, *P *< 0.0001; Figure 2d). The puromycin‐based approach for quantifying MPS did not capture the responses of hMMTs to the anabolic stimuli and produced data with large variability (means ± SD and *P*‐values are presented in Appendix Table A3; Figure 2e,f). This aligns with a linear regression showing a weak and statistically insignificant relationship between puromycin incorporation and sarcoplasmic FSR (*R* ^2^ = 0.098, *m* = −0.759, *P = *0.168; Figure 2g). Thus, using stable isotope tracers to measure MPS, we observed that acute amino acid‐stimulated protein synthesis in hMMTs is not enhanced by contractile stimulation or combined ketone treatment. There were no differences in puromycin‐derived measurements of MPS (main effect *P = *0.870), further emphasizing the utility of the tracer‐based approach for experiments utilizing longer treatment periods.

**FIGURE 2:**
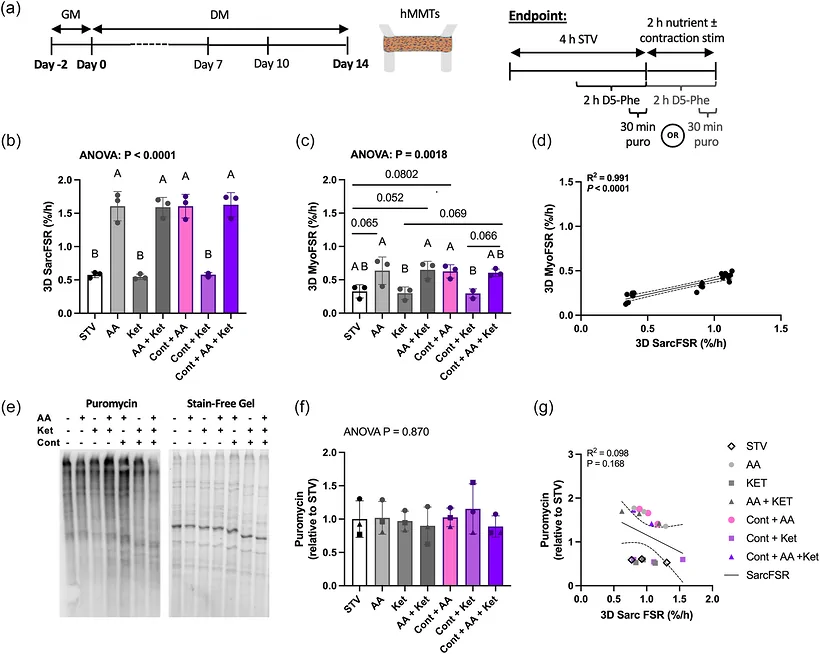
Stable isotope‐ versus puromycin‐derived measurements of nutrient and contraction stimulated muscle protein synthesis (MPS) in human skeletal muscle microtissues (hMMTs). (a) Schematic overview of the timeline for hMMT culture and endpoint MPS measurements. hMMTs were cultured in after‐seeding growth medium (GM) for 2 days, followed by differentiation medium for 14 days. MPS was measured via incorporation of l‐[ring‐^2^H_5_]phenylalanine (D5‐Phe) and puromycin during anabolic knockdown (starved [STV]), or stimulation with amino acids (AA), ketones (Ket) or both (AA + Ket) following knockdown, with or without an accompanying electrical contraction (Cont) regime prior to treatment. (b, c) Sarcoplasmic (Sarc; b) and myofibrillar (Myo; c) fractional synthetic rates (FSR) measured via D5‐Phe incorporation into sarcoplasmic and myofibrillar proteins, respectively. (d) Linear regression modelling the relationship between sarcoplasmic and myofibrillar FSR. (e) Representative image of western blot analysis for puromycin alongside the corresponding stain‐free total protein image. (f) Quantification of puromycin‐labelled peptides normalized to STV. (g) Linear regression modelling the relationship between sarcoplasmic FSR and puromycin incorporation. *n* = 5–7 hMMTs pooled per condition across *N* = 3 independent experiments. Graphs display means ± SD. Circular, triangular and square dots represent values from the first, second and third biological replicate, respectively. Statistical significance was determined using one‐way ANOVA followed by Tukey's post hoc test; different letters denote statistically different conditions (*P < *0.05) and *P*‐values greater than 0.05 but less than 0.10 are shown.

### 3.3 Myofibrillar protein fraction of hMMTs exhibits greater stability than in 2D myotubes

We performed linear regression analyses to assess the relationships between tracer‐derived sarcoplasmic FSR (Figure 3a), myofibrillar FSR (Figure 3b) and puromycin incorporation (Figure 3c) in 2D myotubes and 3D hMMTs under nutrient stimulation. Both sarcoplasmic FSR and myofibrillar FSR were highly correlated across models (*R* ^2^ = 0.960 SarcFSR; and *R* ^2^ = 0.809, MyoFSR). The sarcoplasmic FSR regression slope (*m* = 1.009, *P *< 0.0001) suggests similar nutrient‐stimulated responses in the sarcoplasmic protein fraction of myotubes and hMMTs, whereas the myofibrillar FSR regression slope (*m* = 0.494, *P *< 0.0001) indicates reduced responsiveness of the myofibrillar fraction of hMMTs. Accordingly, the sarcoplasmic‐to‐myofibrillar FSR ratio across nutrient stimulation conditions was higher in hMMTs (2.326 ± 0.357) compared to myotubes (1.239 ± 0.085; *P *< 0.0001; Welch's *t*‐test, Table 1). Puromycin incorporation did not show a significant correlation (*R* ^2^ = 0.072, *P = *0.398) across models. Comparison of tracer‐based measurement of MPS in 2D myotubes and hMMTs reflects the distinct biological properties of these skeletal muscle models, with the myofibrillar fraction in hMMTs exhibiting greater stability than that in 2D myotubes. Similar results were obtained when the contractile stimulus group was excluded from analyses (Table 2).

**FIGURE 3:**
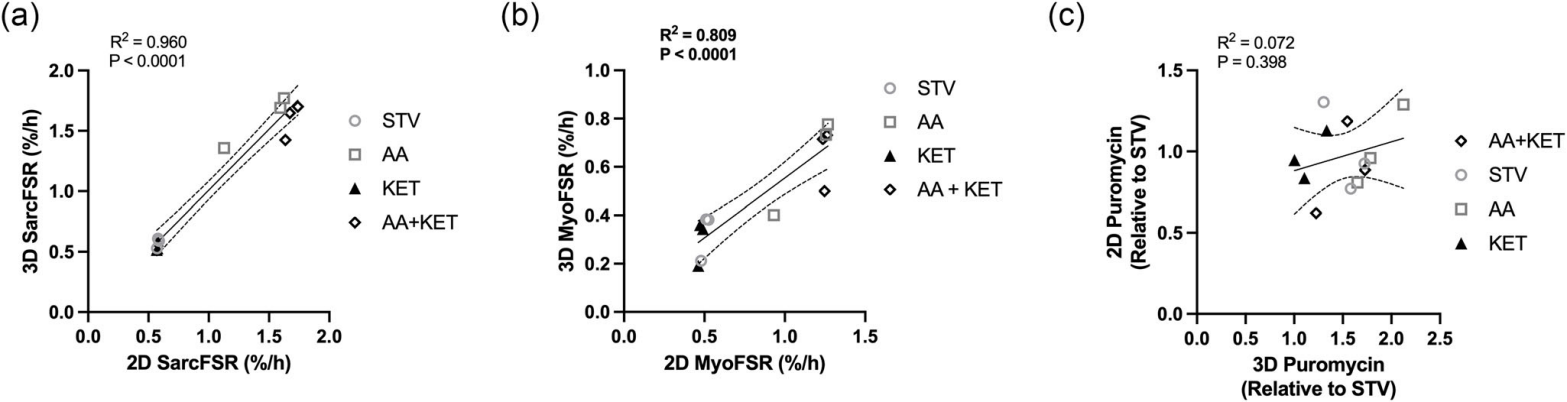
Comparison of stable isotope‐ and puromycin‐derived measurements of nutrient stimulated protein synthesis in 2D myotubes versus 3D human skeletal muscle microtissues (hMMTs). Linear regressions illustrating the relationships for measurements of tracer‐derived sarcoplasmic fractional synthetic rate (Sarc FSR; a), myofibrillar fractional synthetic rate (Myo FSR; b), and puromycin incorporation (c) in 2D myotubes versus 3D hMMTs. Measurements for starved (STV), amino acid (AA), ketone (Ket), and combined amino acid and ketone (AA + Ket) stimulation conditions were used in the analysis. Data points represent the mean of *N* = 3 biological replicates.

**TABLE 1:** Sarcoplasmic‐to‐myofibrillar fractional synthetic rate (FSR) ratio across stimulation conditions in 2D myotubes and 3D hMMTs.

Culture	Stimulation condition	Sarcoplasmic‐to‐myofibrillar FSR ratio	Mean ratio ± SD	*P*
2D myotubes	STV	1.15	1.239 ± 0.085	<0.0001
	AA	1.26		
	Ket	1.21		
	AA + Ket	1.35		
3D hMMTs	STV	1.87	2.326 ± 0.357	
	AA	2.66		
	Ket	1.96		
	AA + Ket	2.49		
	Cont + AA	2.58		
	Cont + Ket	2.02		
	Cont + AA + Ket	2.69		

*Note*: Compared using a Welch's *t*‐test. Abbreviations: AA, amino acid; Ket, ketone; STV, starved.

**TABLE 2:** Sarcoplasmic‐to‐myofibrillar fractional synthetic rate (FSR) ratio across stimulation conditions in 2D myotubes and 3D hMMTs without contractile stimulation conditions.

Culture	Stimulation condition	Sarcoplasmic‐to‐myofibrillar FSR ratio	Mean ratio ± SD	*P*
2D myotubes	STV	1.15	1.239 ± 0.085	0.0116
	AA	1.26		
	Ket	1.21		
	AA + Ket	1.35		
3D hMMTs	STV	1.87	2.248 ± 0.386	
	AA	2.66		
	Ket	1.96		
	AA + Ket	2.49		
	Cont + AA	2.58	Contractile stimulation conditions not included	
	Cont + Ket	2.02		
	Cont + AA + Ket	2.69		

*Note*: Compared using a Welch's *t*‐test.

Abbreviations: AA, amino acid; Ket, ketone; STV, starved.

## 4 DISCUSION

In the present study, we demonstrate that stable isotope amino acid tracer was more sensitive at detecting acute nutrient‐induced changes in protein synthesis than puromycin incorporation. Stable isotopes enabled fraction‐specific measurements of protein synthesis in skeletal muscle in vitro and captured anabolic responses that puromycin failed to detect. Although there was a high level of agreement between the responses of 2D and 3D skeletal muscle models, 3D hMMTs displayed a sarcoplasmic‐to‐myofibrillar FSR ratio of ∼2:1 across conditions (Figure 2), compared to a ratio closer to ∼1:1 in 2D myotubes (Figure 1). This finding can be reinforced in future studies by evaluating multiple donor cell lines. Notably, the ratio ∼2:1 observed for hMMTs closely resembles that of native human skeletal muscle (Abou Sawan et al., 2021; Moore et al., 2009), demonstrating the ability of bioengineered models to better recapitulate the fraction‐specific MPS responses characteristic of human muscle. However, the ketones β‐hydroxybutyrate and lithium acetoacetate did not enhance muscle protein synthesis, either in the absence of amino acids – likely due to the lack of a basal amino acid pool found in vitro – or in the presence of supraphysiological concentrations of essential amino acids. Furthermore, contractile stimulation did not potentiate the response to amino acid sufficiency during the acute post‐stimulation period, suggesting that a longer duration or different electrical pulse parameters may be required to observe an effect. Overall, our results highlight the value of the tracer‐based approach in providing insights into protein synthesis not revealed by puromycin.

### 4.1 Limitations of puromycin‐based approach likely explain lack of observed differences

Puromycin incorporation is a convenient and cost‐effective method to measure nascent peptide synthesis, but it has several limitations that affect its utility. Unlike stable isotope tracers, puromycin is neither chemically nor functionally identical to naturally occurring amino acids. As a potent inhibitor of translation, puromycin terminates protein synthesis upon incorporation into elongating peptide chains, making its integration into functional proteins highly unlikely (Aviner, 2020). Furthermore, puromycin assays are performed by immunoblotting the soluble sarcoplasmic fraction of cell lysates. Although this approach only assesses the sarcoplasmic fraction, it is probably more reflective of a ‘mixed’ protein fraction, because puromycin‐tagged truncated proteins likely fail to reach their intended cellular compartments and remain in the sarcoplasm (Enam et al., 2020). The inherently lower precision of immunoblotting, when compared to LC‐MS/MS (Aebersold et al., 2013), likely contributes to the greater variability observed between biological replicates in our puromycin‐based measurements. Indeed, our stable isotope‐based approach had less variability between replicates and therefore is likely to be superior for capturing small differences between conditions. Additionally, puromycin labelling does not accurately measure mRNA translation rates in energetically challenged cells. For example, in glucose‐ and amino acid‐starved HEK293 cells, puromycin incorporation showed a significantly smaller reduction in protein synthesis compared to radiolabelled amino acid ([^35^S]methionine) incorporation (Marciano et al., 2018). This suggests that puromycin may underestimate the extent of translational inhibition under nutrient‐deprived conditions, such as in the 2D myotubes and hMMTs in our study following anabolic knockdown. Another key limitation is that the shorter 30‐min incorporation period used for the puromycin, compared to the 2 h for tracer, may capture distinct protein synthesis trends. Short incorporation times preferentially bias measurement towards proteins with rapid turnover rates (Miller et al., 2015), thereby under‐representing proteins with slower turnover dynamics. It is worth noting others have used puromycin for longer (4 h) incorporation periods in C2C12 myotubes without observing a negative impact (Carson et al., 2018). We elected to use puromycin for 30 min as others have done (Goodman et al., 2011; Ham et al., 2014) to compare against standard practice. However longer periods might better reflect tracer‐derived measures of MPS. These limitations highlight the need for cautious interpretation of puromycin‐derived measurements of protein synthesis and highlight how tracer‐based approaches address some of these shortcomings.

### 4.2 Absence of ketone‐induced increase in MPS likely due to insufficient amino acid availability

Ketone supplementation with β‐hydroxybutyrate and lithium acetoacetate in amino acid‐free DMEM failed to stimulate protein synthesis in 2D myotubes or hMMTs relative to control amino acid‐free DMEM following anabolic knockdown. In contrast, ketone monoester supplementation in fasted human skeletal muscle increased MPS to a level comparable to a small bolus (10 g) of whey protein (Hannaian et al., 2024). This disparity suggests that while ketones can stimulate MPS in vivo, they likely require a basal concentration of amino acids as substrates for protein translation. In vivo, human skeletal muscle in the overnight fasted (post‐absorptive) state maintains amino acids levels sufficient to support protein synthesis, whereas in vitro models lack this physiological baseline. Moreover, our studies demonstrated that ketones did not enhance protein synthesis in the presence of supraphysiological amino acid concentrations found in DMEM in either 2D myotubes or hMMTs. This finding aligns with evidence from human skeletal muscle, where ketone monoester supplementation did not exert additive effects when combined with whey protein ingestion (Hannaian et al., 2024). Future work should investigate ketone supplementation in medium containing physiological concentrations of amino acids that more closely resemble human serum composition.

### 4.3 Contraction‐mediated increases in MPS likely missed due to short measurement duration

While amino acid provision increased MPS in hMMTs, contraction did not provide any additional enhancement. This differs from in vivo human data, where resistance exercise (i.e., contraction) combined with protein ingestion induces a greater stimulation of MPS than protein alone, primarily due to elevated myofibrillar protein synthesis as early as 3 h and for up to 24 h after (Burd et al., 2011; Moore et al., 2009). Given that our labelling period was limited to 2 h post‐stimulation, the full potentiation effect of contraction may not have been captured. Indeed, previous work using cyclic stretch of monolater L6 myotubes has demonstrated an acute reduction in MyoFSR which returned to baseline 2.5 h post‐stretch (Atherton et al., 2009). Alternatively, our contraction parameters may have been insufficient to induce anabolism in the present study. Indeed, a similar contraction regime on hMMTs constructed using immortalized human myoblasts elicited no change in myosin heavy chain protein levels by western blot (Lad et al., 2025), whereas evidence of anabolism was observed in hMMTs following 7 days of intermittent electrical stimulation regimes (Khodabukus et al., 2019). Future work using 3D models should explore several stimulation parameters combined with stable isotope‐derived measurements to further explore this experimental space. Overall, future studies should extend the MPS measurement window and include a contraction‐only control (i.e. without nutrient manipulation), and several stimulation parameters to establish whether hMMTs exhibit increased myofibrillar MPS in response to the synergistic effect of contraction and amino acid provision, but not contraction alone, as is observed in vivo.

### 4.4 Conclusions

Overall, we demonstrate the utility of stable isotope tracers for measuring protein synthesis in 2D and 3D human skeletal muscle culture models. Our findings indicate hMMTs generated in the MyoTACTIC platform better recapitulate human physiological responses to nutrient stimulation compared to 2D myotubes derived from the same primary cell line. Future work in this space should prioritize use of physiologically relevant skeletal muscle models and utilize stable isotope‐derived measurements to enhance the accuracy of muscle protein synthesis assessments.

## AUTHOR CONTRIBUTIONS

Cassidy T. Tinline‐Goodfellow and Yekaterina Tiper were responsible for the acquisition and analysis of the data presented in this work. All authors contributed to the study's conception and design, and data interpretation. Cassidy T. Tinline‐Goodfellow and Yekaterina Tiper drafted the manuscript. All authors revised it critically for important intellectual content. The final version of the manuscript has been approved by all authors, who agree to be accountable for all aspects of the work in ensuring that questions related to the accuracy or integrity of any part of the work are appropriately investigated and resolved. All persons designated as authors qualify for authorship, and all those who qualify for authorship are listed.

## CONFLICT OF INTEREST

None declared.

## GENERATIVE AI STATEMENT

Generative AI (ChatGPT V4.0, OpenAI) was used to refine writing and grammar but was not used to analyse or interpret data.

## Data Availability

The data that support the findings of this study are available from the corresponding authors upon reasonable request.

## 1

**TABLE A1:** Composition of media and solutions used for human primary myoblast expansion, 2D myotube differentiation, hMMT culture and endpoint anabolic stimulation.

Medium/solution	Composition
Rat tail collagen coating solution for myoblast passaging	∼16 µg/mL rat tail collagen I (cat. no. A1048301, Thermo Fisher Scientific) in 0.1 M acetic acid solution prepared with dH_2_O
Myoblast growth medium	Ham's F‐10 nutrient mix (cat. no. 318‐050‐CL, Wisent Bioproducts), 20% FBS (cat. no. 12483020, Thermo Fisher Scientific), 5 ng/mL bFGF (cat. no. 11343625, ImmunoTools), 1% P/S (cat. no. 15140122, Thermo Fisher Scientific)
Rat tail collagen coating solution for 2D differentiation	∼50 µg/mL rat tail collagen I in 0.1 M acetic acid solution prepared with dH_2_O
2D differentiation medium	High glucose (4.5 g/L) DMEM (cat. no. 11995065, Thermo Fisher Scientific), 2% HS (cat. no. 16050114, Thermo Fisher Scientific), 10 µg/mL insulin (cat. no. I6634, Sigma‐Aldrich), 1% P/S
Fibrinogen stock solution	10 mg/mL fibrinogen in 0.9% (w/v) NaCl solution prepared with dH_2_O
Hydrogel master mix	High glucose DMEM (40% v/v), 4 mg/mL fibrinogen (40% v/v), Geltrex™ (20% v/v; cat. no. A1413202, Thermo Fisher Scientific)
After‐seeding growth medium	Ham's F‐10 nutrient mix, 20% FBS, 1.5 mg/mL ACA (3% v/v; cat. no. A2504, Sigma‐Aldrich), 1% P/S
3D differentiation medium	High glucose DMEM, 2% HS, 2 mg/mL ACA (4% v/v), 10 µg/mL insulin, 1% P/S
Anabolic knockdown medium (i.e. AA‐starvation medium)	Low glucose (1.0 g/L) DMEM w/o amino acids (cat. no. D9800‐13, United States Biological Corporation), 3.5 g/L d‐glucose (bringing total glucose up to 4.5 g/L), 1 mM sodium pyruvate (cat. no. 11360070, Thermo Fisher Scientific), 3.7 g/L sodium bicarbonate, 2 mg/mL ACA (4% v/v), 1% P/S
Amino acid stimulation medium	High glucose DMEM, 2 mg/mL ACA (4% v/v), 1% P/S
Ketone stimulation medium	Low glucose DMEM w/o amino acids, 3.5 g/L d‐glucose (4.5 g/L total), 1 mM sodium pyruvate, 3.7 g/L sodium bicarbonate, 4 mM 3‐hydroxybutyrate, 1.4 mM lithium acetoacetate, 2 mg/mL ACA (4% v/v), 1% P/S
Amino acid and ketone stimulation medium	High glucose DMEM, 4 mM 3‐hydroxybutyrate, 1.4 mM lithium acetoacetate, 2 mg/mL ACA (4% v/v), 1% P/S

*Note*: ImmunoTools: Friesoythe, Germany; Sigma‐Aldrich: St Louis, MO, USA; Thermo Fisher Scientific: Waltham, MA, USA; United States Biological Corporation: Salem, MA, USA; Wisent Bioproducts: Saint‐Jean Baptiste, QC, Canada.

Abbreviations: ACA, 6‐aminocaproic acid; bFGF, basic fibroblast growth factor; DMEM, Dulbecco's modified Eagle's medium; FBS, fetal bovine serum; HS, horse serum; P/S, penicillin–streptomycin.

**TABLE A2:** Pooled sample counts, means ± standard deviation (SD), statistical tests and significance values for measurements of protein synthesis in 2D studies.

Data presented	Figure	Sample	Mean ± SD	*P* (relative to control, STV)*
Sarc FSR (%/h)	1b	STV	0.579 ± 0.008	N/A
		AA	1.449 ± 0.278	0.0003
		Ket	0.573 ± 0.004	>0.999
		AA + Ket	1.683 ± 0.052	<0.0001
Myo FSR (%/h)	1c	STV	0.505 ± 0.024	N/A
		AA	1.152 ± 0.191	0.0002
		Ket	0.476 ± 0.015	0.981
		AA + Ket	1.249 ± 0.012	<0.0001
Puromycin (relative to STV)	1f	STV	1.000 ± 0.139	N/A
		AA	1.206 ± 0.159	*P*0.366
		Ket	0.747 ± 0.110	*P*0.220
		AA + Ket	0.977 ± 0.165	0.997

*Notes*: Pooled sample counts: *n* = 3 wells pooled from a 6‐well plate, averaged over *N* = 3 independent experiments. Statistical test: one‐way ANOVA + Tukey's test.

Abbreviations: AA, amino acid; FSR, fractional synthetic rate; Ket, ketone; Myo, myofibrillar; Sarc, sarcoplasmic; STV, starved.

* Reported *P*‐values are multiplicity adjusted values (Tukey's) for each comparison.

**TABLE A3:** Pooled sample counts, means ± standard deviation (SD), statistical tests and significance values for measurements of protein synthesis in 3D studies.

Data presented	Figure	Sample	Mean ± SD	*P* (relative to control, STV)*
Sarc FSR (%/h)	2b	STV	0.577 ± 0.042	N/A
		AA	1.606 ± 0.22	<0.0001
		Ket	0.546 ± 0.041	>0.999
		AA + Ket	1.591 ± 0.147	<0.0001
		Cont + AA	1.607 ± 0.178	<0.0001
		Cont + Ket	0.58 ± 0.031	>0.999
		Cont + AA + Ket	1.628 ± 0.181	<0.0001
Myo FSR (%/h)	2c	STV	0.326 ± 0.099	N/A
		AA	0.637 ± 0.205	0.065
		Ket	0.299 ± 0.093	>0.999
		AA + Ket	0.649 ± 0.129	0.052
		Cont + AA	0.625 ± 0.1	0.080
		Cont + Ket	0.296 ± 0.068	0.999
		Cont + AA + Ket	0.606 ± 0.056	0.114
Puromycin (relative to STV)	2f	STV	1 ± 0.274	N/A
		AA	1.019 ± 0.246	>0.999
		Ket	0.971 ± 0.148	>0.999
		AA + Ket	0.898 ± 0.283	0.983
		Cont + AA	1.026 ± 0.14	>0.999
		Cont + Ket	1.154 ± 0.375	0.985
		Cont + AA + Ket	0.889 ± 0.16	0.997

*Notes*: Pooled sample counts: *n* = 5–7 pooled hMMTs, averaged over *N* = 3 independent experiments. Statistical test: one‐way ANOVA + Tukey's test.

Abbreviations: AA, amino acid; Cont, contraction; FSR, fractional synthetic rate; Ket, ketone; Myo, myofibrillar; Sarc, sarcoplasmic; STV, starved.

* Reported *P*‐values are multiplicity adjusted values for each comparison.

**TABLE A4:** The *x*‐ and *y*‐axis metrics, *F*‐statistic, significance value, slope and coefficient of determination for each linear regression model.

Metric		Figure	*F*	*P*	Slope (*m*)	*R* ^2^
*x*‐axis	*y*‐axis					
2D Sarc FSR	2D Myo FSR	1d	*F*(1,10) = 1187	<0.0001	0.707	0.992
2D Sarc FSR	2D Puromycin	1g	*F*(1,10) = 1.671	0.225	0.631	0.143
3D Sarc FSR	3D Myo FSR	2d	*F*(1,19) = 111.2	<0.0001	0.326	0.854
3D Sarc FSR	3D Puromycin	2g	*F*(1,19) = 2.057	0.168	−0.759	0.098
2D Sarc FSR	3D Sarc FSR	3a	*F*(1,10) = 236.8	<0.0001	1.009	0.960
2D Myo FSR	3D Myo FSR	3b	*F*(1,10) = 42.36	<0.0001	0.494	0.809
3D Puromycin	2D Puromycin	3c	*F*(1,10) = 0.781	0.398	0.178	0.072

Abbreviations: FSR, fractional synthetic rate; Myo, myofibrillar; Sarc, sarcoplasmic.
